# Investigating the Function of Play Bows in Dog and Wolf Puppies (*Canis lupus familiaris*, *Canis lupus occidentalis*)

**DOI:** 10.1371/journal.pone.0168570

**Published:** 2016-12-29

**Authors:** Sarah-Elizabeth Byosiere, Julia Espinosa, Sarah Marshall-Pescini, Barbara Smuts, Friederike Range

**Affiliations:** 1 Department of Psychology, University of Michigan, Ann Arbor, United States of America; 2 Comparative Cognition, Messerli Research Institute, University of Veterinary Medicine, Vienna, Medical University of Vienna, University of Vienna, Vienna, Austria; 3 Wolf Science Centre, Ernstbrunn, Austria; University of Lethbridge, CANADA

## Abstract

Animals utilize behavioral signals across a range of different contexts in order to communicate with others and produce probable behavioral outcomes. During play animals frequently adopt action patterns used in other contexts. Researchers have therefore hypothesized that play signals have evolved to clarify communicative intent. One highly stereotyped play signal is the canid play bow, but its function remains contested. In order to clarify how canid puppies use play bows, we used data on play bows in immature wolves (ages 2.7–7.8 months) and dogs (ages 2 to 5 months) to test hypotheses evaluated in a previous study of adult dogs. We found that young dogs used play bows similarly to adult dogs; play bows most often occurred after a brief pause in play followed by complementary highly active play states. However, while the relative number of play bows and total observation time was similar between dog and wolf puppies, wolves did not follow this behavioral pattern, as play bows were unsuccessful in eliciting further play activity by the partner. While some similarities for the function of play bows in dog and wolf puppies were documented, it appears that play bows may function differently in wolf puppies in regards to re-initiating play.

## Introduction

Communication can be defined as a multifaceted exchange between senders and receivers each with their own targets or goals [1]. Within this exchange an individual performs a signal in order to induce the receiver of said signal to perform a probable pattern [2]. These signals may be “behavioral, physiological, or morphological characteristics fashioned or maintained by natural selection [as] they convey information to other organisms” [3, p. 385].

In non-human animals (hereafter animals), signals are often assumed to represent simple single traits [4]. This oversimplification ignores the fact that signals often occur in complex contexts and are imperative in animal communication. Individuals must signal that they wish to initiate an interaction and then negotiate the nature of it [5,6]. This means that senders must choose an appropriate signal(s) based on context-specific information to ensure a successful outcome [7]. Furthermore, multiple single signal components may be combined into a multimodal signal, thereby inducing additional behavioral outcomes (for examples see [8]). Considering the variety of mediums (such as visual, auditory, and olfactory), intensities, frequencies, and situational contexts in which signals can occur, proper utilization is highly complex.

While signals are performed across a range of contexts, their function within play behavior is particularly fascinating [9]. Within playful interactions, action patterns common to other contexts such as aggressive and sexual bouts are often performed [6,10–12]. Therefore, these generalizable action patterns may, on a surface level, appear fundamentally identical while the communicative intent behind them may vary drastically [13]. Researchers have hypothesized that animals discriminate between these contexts and maintain a playful atmosphere through the use of *play signals*.

Play signals have typically been thought to communicate playful intentions in order to commence, continue, and recommence social play [5,14,15]. Thus, they may serve as social mediators, or ways for the signaler to convey his/her intentions in a manner easily interpretable for the receiver [16]. They can co-occur as modifiers of other behaviors, such as an open mouth display while charging, or they can be specific actions, like exaggerated bouncy movements interspersed among other behaviors. Play signals may vary or even evolve within or across structured play bouts in a way to redefine the appropriate “rules” of play [9,17]. Consequently, maintaining a playful bout not only requires sophisticated communicative skill, but also communicative effort, improvisation, strategic timing, creativity, and the capacity to cope with unexpected events or unpredictable partner reactions [9,18].

Canids, such as dogs and wolves, are unusual in that they exhibit high frequencies of play behaviors even as adults (for a review of dog play see [19,20]). Furthermore, they exhibit one of the most stereotyped and easily recognizable carnivore play signals, the play bow [21]. This high-rump crouch position occurs when the forelegs of an individual are bent, often in a lying down position, while the hindquarters remain elevated [22]. This distinctive position is consistently found within dog play as well as in other closely related species such as coyotes, wolves, foxes and even lions [22,23]. Play bows are considered to be visual signals. Horowitz [11], and Byosiere et al. [24] found that during adult dog dyadic play, play bows as well as other play signals requiring visual attention, nearly always occurred when the pair faced one another after a brief pause (simillar findings have also been observed in [25]). In addition, non-visual “attention-getting” behaviors, such as vocalizations and touching, were more common when play partners were facing away, and matched the receiver’s degree of inattentiveness.

While the play bow is a widespread and easily recognizable signal, its function within play has rarely been addressed. Bekoff [26] found that play bows were more likely to occur in association with behaviors that could potentially be misinterpreted as aggression, particularly bite-shakes. In contrast, Pellis and Pellis [16] suggested that play bows may in fact not be play signals at all, positing that instead, this posture could function as a strategic position that allows the bower to launch a mock-attack on the play partner and/or to better escape from him/her. Moreover, play bows may help strengthen bonds between dogs that share common motivations. Palagi et al. [27] found that during dyadic dog play, both the open mouth display and play bow often elicited the same behavior in the partner immediately afterward, thus suggesting that rapid mimicry may indicate shared positive emotions and facilitate behavioral coordination.

Recently, Byosiere et al. [24] assessed these hypotheses in a sample of adult pet dogs. They found no evidence to suggest that play bows were used to clarify easily misinterpretable behaviors, or that play bows function as strategic positions. Instead they reported that the most common behavior for both the bower and the partner before the play bow was a stationary position, and that after the bow dogs most commonly showed active behaviors such as mutual rear-ups and complimentary runaway/chase sequences. Therefore, the authors concluded that one important function of the play bow is to re-initiate play after a pause [24].

Due to the paucity of research and lack of consensus about the function of play bows in dogs and other canids, the current study adopted a comparative approach to further clarify the purpose of this highly stereotyped play signal. Specifically, we aimed to investigate whether the behavioral patterns observed around play bow signaling in adult dogs developed similarly in both dog and wolf puppies. In doing so we expand on the study by Byosiere et al. [24] that assessed a variety of proposed functions such as whether play bows function as visual signals [11,24], to clarify intentions [26], to resume play, to occupy a better strategic position [16], or to synchronize behavioral actions [27,28].

### Hypotheses and Predictions

To further examine the function of play bows in wolf and dog puppies, behaviors occurring just before and just after the play bow for both the play bower and the play partner were analyzed (following the methods of [24]; see Table 1 for a summary of supported hypotheses). The following hypotheses derive from the studies described in the introduction and predict outcomes that may not be mutually exclusive (hypothesis 1, 2 and 5), while others may be (hypotheses 2 and 3).

Hypothesis 1: Play bows as visual signals
  1. If play bows function as visual signals, play bows should be limited to times when the bower and partner are within one another’s visual field (S1 Table).
  2. If play bows function as visual signals, play bows performed when the players are not within one another’s visual fields should occur in association with attention-getting behaviors, as defined by Horowitz (2009).
Hypothesis 2: Clarification of easily misinterpretable behaviors
  If play bows function to clarify behaviors by the bower that are most susceptible to misinterpretation, the bower should perform more offensive behaviors than the partner before and/or after play bowing.
Hypothesis 3: Attacking and escaping the play partner
  1. If play bows function to obtain an optimal position to better ‘attack’ the play partner, the bower should perform more offensive behaviors after the play bow than before.
  2. If play bows function to obtain an optimal position to better ‘escape’ the play partner, the bower should perform more vulnerable/escape behaviors, such as runaway (for definitions see S1 File), after the play bow than before.
Hypothesis 4: Re-initiation of play
  If play bows function to reinitiate play, both the bower and the partner should perform more pauses and passive non-play behaviors before the play bow than after.
Hypothesis 5: Play bow synchronization
  If play bows function to help synchronize play behaviors, the bower and the partner should perform more synchronous behaviors after the play bow than before.

**Table 1:** Summary of evidence for hypotheses 1–5 in adult dogs from Byosiere et al., (2016).

Hypothesis	Adult Dogs
1: Play bows as visual signals	Yes
2: Clarification of easily misinterpretable behaviors	No
3a: Attacking the play partner	No
3b Escaping from the play partner	Maybe
4: Re-initiation of play	Yes
5: Play bow synchronization	Yes

## Materials and Methods

### Ethical Statement

No special permission for use of animals (wolves and dogs) in such observational studies is required in Austria (Tierversuchsgesetz 2012- TVG 2012). The relevant committee that allows running research without special permissions regarding animals is: Tierversuchs-kommission am Bundesministerium für Wissenschaft und Forschung (Austria).

### Subjects

Dog and wolf puppies were raised in the same way, hand-reared in peer groups, and bottle-fed/hand-fed by humans from 10 days of age to 4 months. The dogs were mongrels born in Hungarian animal shelters or at the Wolf Science Center in Ernstbrunn, Austria. All wolves were born in captivity. Dogs observed in 2014 were born at the center, and spent at least four hours per day with a trainer and other puppies (without the mother) (see [29] for additional information). Both species were kept in a similar manner and therefore have the same life experiences in order to compare the two without fundamental differences in ontogeny.

Dog subjects (*Canis lupus familiaris*) consisted of 10 dogs. All play interactions involved dog puppies of 75 to 140 days (2 to 5 months). See Table 2 for demographic and relatedness information for dogs. Wolf subjects (*Canis lupus occidentalis*) consisted of 15 timber wolves. All play interactions involved wolves of 85 to 1254 days (2.7 months to 3.4 years), however only play bows performed by wolf puppies aged 85 days to 239 days (2.7 to 7.8 months) were analyzed. Therefore a play partner could be an older wolf, however no play bow performed by an older wolf was included in the analysis.

**Table 2:** Demographic data for dog puppies sampled.

Name	Sex	Siblings	Puppy Pack
Banzai	M	Enzi, Panya, Pepeo	Pack A (2014)
Enzi	M	Banzai, Panya, Pepeo	Pack A (2014)
Gombo	M	Hiari, Imara, Sahibu	Pack A (2014)
Hiari	M	Gombo, Imara, Sahibu	Pack A (2014)
Imara	F	Gombo, Hiari, Sahibu	Pack A (2014)
Nia	F		Pack B (2011)
Kali	F		Pack B (2011)
Panya	F	Banzai, Enzi, Pepeo	Pack A (2014)
Pepeo	M	Banzai, Enzi, Panya	Pack A (2014)
Sahibu	F	Gombo, Hiari, Imara	Pack A (2014)

Additional analyses were conducted and revealed no significant difference in the results when the adults were removed as partners from the sample.

See Table 3 for demographic and relatedness information for wolves. One set of wolf puppies was observed in 2009 and one in 2012 [29]. Each pack consisted of six wolf puppies with both kin and non-kin (no more than two individuals from one litter per pack). Wolf puppies lived in a single pack for the first four months of life. Next, they were introduced into already established packs of adult wolves (mixed pack). In 2009, all six puppies were integrated into a previously established group of three adult wolves. In 2012, the six puppies were separated in twos and integrated into each of the three mixed packs (Table 3).

**Table 3:** Demographic data for Timber wolf puppies sampled.

Name	Sex	Siblings	Birthplace	Puppy Pack	Adult Pack
Amarok	M	Tala	Minnesota Wildlife Connection	Pack B (2012)	Pack C
Apache	M	Cherokee	Zoo Basel	Pack A (2009)	Pack A
Aragorn	M	Shima	Herberstein Zoo		Pack A (2009), Pack D (2012)
Cherokee	M	Apache	Zoo Basel	Pack A (2009)	Pack A
Chitto	M	Una	Minnesota Wildlife Connection	Pack B (2012)	Pack D
Geronimo	M	Yukon	Triple D Farm, Montana USA	Pack A (2009)	Pack A
Kay	F	Wamblee	Haliburton Forest Canada	PackB (2012)	Pack C
Kaspar	M		Herberstein Zoo		Pack A (2009), Pack D (2012)
Nanuk	M		Triple D Farm, Montana USA	Pack A (2009)	Pack A
Shima	F	Aragorn	Herberstein Zoo		Pack A (2009), Pack D (2012)
Tala	F	Amarok	Minnesota Wildlife Connection	Pack B (2012)	Pack D
Tatonga	F		Triple D Farm, Montana USA	Pack A (2009)	Pack A
Una	F	Chitto	Minnesota Wildlife Connection	Pack B (2012)	Pack B
Wamblee	M	Kay	Haliburton Forest Canada	Pack B (2012)	Pack B
Yukon	F	Geronimo	Triple D Farm, Montana USA	Pack A (2009)	Pack A

### Data Collection and Video Coding

Dyadic play bouts were videotaped for research purposes during 2009 and 2012 for wolf puppies, and 2011 and 2014 for dog puppies by researchers at the Wolf Science Center as part of a study on wolf and dog play and social behavior. Videos coded for the current study were taken from a database of already recorded videos. Permission from management at the Wolf Science Center was obtained to review and analyze previously recorded videos. Some of these videos and data have been analyzed and published (for descriptive information about these videos see [29]). Filming for these interactions took place in the animal’s home enclosures, which were large, fenced outdoor areas; elevated areas used for shelters, as well as trees, brush, and occasionally fallen tree trunks. Video recordings analyzed for this study did not include any feeding times. All observations were distributed throughout the day, from roughly 0600 to 2000 hours. Twenty hours and 39 minutes of video recordings were analyzed for the dog sample, and 16 hours and 53 minutes of video recordings were analyzed for the wolf sample. Videos were recorded on 62 different days for the dogs and 40 for the wolves. The first two authors coded the videos; both had previously reached 90% agreement using this coding protocol (see [24] for a comprehensive review of the methods).

Data was coded in a way so that only the immediate behavior both before and after the play bow were coded for the bower and the partner. Our rationale was that this was the most direct way to test the various hypotheses and to compare how bower and partner behavior may have changed as a result of the play bow. Play bows were only coded when they occurred during a play bout (defined below), not when they were used to initiate a play bout, and when they met specific and detailed criteria involving both the type of movement and duration. Play bows were defined as beginning when an individual’s front legs began to bend, and concluding when an individual fully extended the elbows back to an upright position, lay down, or adopted a new position in which the rear was not elevated above the front end. Play bows had to last at least 1/3 of a second in order to be coded as such [24]. Dyadic play bouts were coded when play continued for at least 15 seconds. The play bout was considered over after a minute without either individual in a dyad showing any of the play behaviors listed in S1 File. We recorded only instances in which play bows occurred during dyadic play bouts. If a third (or fourth, fifth) individual interrupted the bout for more than 15 seconds, the dyadic play bout was considered to be over.

During a play bow, we coded the pair as facing one another when each was within the other’s visual field (S1 Table). Furthermore, behaviors occurring immediately before and immediately after play bows by both the bower and partner were coded (see S1 File for the ethogram). These play behaviors (from here on termed behavior codes) were divided into five mutually exclusive categories: offensive/dominant, vulnerable/escape, pause, synchronous, and miscellaneous (S1 File) [24]. Offensive/dominant behaviors consisted of mock-attack play behaviors (e.g., tackle, bites; [30], chases/charges and receipt of formal submission (i.e., muzzle licks; [31]). Vulnerable/escape behaviors included self-handicapping behaviors, receiving an offensive behavior (e.g., ‘is tackled’), and running away behaviors. Note that many of the behaviors shown in these two categories were reciprocal; that is, if one dog showed an offensive behavior, such as pushing or tackling the other dog, the partner would be coded as receiving a vulnerable/escape behavior, in this case, being pushed/tackled. Pause behaviors were all behaviors that involved little movement (i.e., the individual took two steps or less) and that lasted at least 1.5 seconds.

Synchronous behaviors included two types of actions that both players commonly performed synchronously (e.g. they displayed very similar or identical behaviors overlapping in time), moving together and rearing up together. Note that we did not include any other behaviors occurring in synchrony within the analysis. However, we have noted the instances in which the bower and the partner performed the same behavior. Many of these instances included relatively stationary behaviors and thus were accounted for in the “pause” behavior category. All other instances in which the bower and partner performed the same behavior occurred at extremely low rates (approximately 2% of all behaviors before, and 1.8% after the bow for dog puppies, and 1.4% before and 2.5% after the bow for wolf puppies). Finally when two play bows given by different individuals overlapped in time, they were considered synchronous bows and were used to evaluate hypothesis #5.

### Data Analysis

To evaluate hypotheses 2 through 5, a generalized linear mixed model (GLMM) [32] was used to analyze the proportion of a particular behavior category (Table 3) relative to all other behavior categories combined (binary model) (for a comprehensive review of the model see [24]). The model contained fixed effects for role (bower *versus* partner) and timing (before *versus* after the bow), and a fixed effect interaction between role and timing. Random effects for individual and dyad were also included. Thus, the proportion of a given behavior (P) was modeled as:

$$\text{Logit}\left({\text{P}}_{\text{tij}}\right)=\beta_{0}+\beta_{1}\text{Role}+\beta_{2}\text{Time}+\beta_{3}\left(\text{Role }\times \text{ Time}\right)+b_{0i}+b_{0ij}$$

Where *b* denotes a random effect, *t* denotes timing, *i* denotes the individual dog, and *j* denotes the dyad. Four binary regressions were conducted comparing one behavior category to all other behavior categories. Results, therefore, represent the change in proportion of a particular behavior category across time (i.e., before or after the bow) and/or as a function of role (i.e. bower or partner). Since 4 comparisons, 2 for role and 2 for time, were used per behavior category analysis, a Bonferonni post-hoc correction of .01 was applied to results for each regression to reduce the chances of a type I error. Convergence criteria were satisfied for each test conducted using the logistic binary regression.

Two-tailed one sample *t*-tests (alpha set at 0.05) were conducted to analyze differences in observed bite behaviors performed by the bower and the partner both before and after the bow.

## Results

For the dog puppies, we observed 136 play bows by 10 dogs in 26 dyads (2 female-female dyads, 12 male-female, and 12 male-male) (Table 4). Play bows occurred at a rate of 10.97% across the observed video time. For the wolf puppies we observed 69 play bows during play by 15 wolves in 25 dyads (4 female-female, 12 male-female dyads and 9 male-male dyads) (Table 5). Play bows occurred at a rate of 6.81% across the observed video time. The number of play bows per dyad in the dog puppies varied from 1 to 16, and 1 to 13 in wolf puppies. For a comprehensive overview of the data output see S2 File.

**Table 4:** Distribution of play bows within and across dog puppy dyads.

Dyad	Dyad Sex	Play Bow Count^1^	Bow Mean^2^	Play Bow Ratio^3^
Banzai/Enzi	M/M	6	4.41	1:5
Banzai/Imara	M/F	4	2.94	2:2
Banzai/Pepeo	M/M	2	2.21	1:0
Banzai/Sahibu	M/M	1	0.74	1:0
Enzi/Gombo	M/M	5	3.68	5:0
Enzi/Hiari	M/M	12	8.82	3:8
Enzi/Imara	M/F	13	9.56	4:9
Enzi/Panya	M/F	7	5.15	3:4
Enzi/Pepeo	M/M	16	11.76	8:8
Enzi/Sahibu	M/M	2	1.47	1:1
Hiari/Banzai	M/M	1	0.74	0:1
Hiari/Imara	M/F	1	0.74	1:0
Hiari/Panya	M/F	6	4.41	4:2
Hiari/Pepeo	M/M	8	5.88	6:2
Imara/Gombo	F/M	1	0.74	1:0
Imara/Pepeo	F/M	12	8.82	9:3
Nia/Kali	F/F	1	0.74	1:0
Panya/Banzai	F/M	3	2.21	3:2
Panya/Gombo	F/M	3	2.21	3:0
Panya/Imara	F/F	1	0.74	1:0
Panya/Pepeo	F/M	3	2.21	3:0
Panya/Sahibu	F/M	10	7.35	7:3
Pepeo/Gombo	M/M	1	0.74	0:1
Pepeo/Sahibu	M/M	13	9.56	7:6
Sahibu/Gombo	M/M	3	2.21	2:1
Sahibu/Imara	M/F	1	0.74	1:0

*Note*:

^1^—The number of play bows performed by members of the dyad.

^2^—The mean of play bows performed by members of the dyad across all play bows for the sample.

^3^—The relative number of play bows within the dyad by individual. The first value corresponds to the number of play bows performed by the first dog in the corresponding “dyad” column. The second value corresponds to the number of play bows performed by the second dog in the corresponding “dyad” column.

**Table 5:** Distribution of play bows within and across wolf puppy dyads.

Dyad	Dyad Sex	Play Bow Count^1^	Bow Mean^2^	Play Bow Ratio^3^
Aragorn/Tatonga	M/F	1	1.45	0:1
Aragorn/Cherokee	M/M	1	1.45	0:1
Geronimo/Cherokee	M/M	1	1.45	0:1
Geronimo/Tatonga	M/F	3	4.35	1:2
Chitto/Kay	M/F	1	1.45	1:0
Tala/Wamblee	F/M	1	1.45	1:0
Tala/Amarok	F/M	2	2.90	2:0
Apache/Cherokee	M/M	13	18.84	4:9
Shima/Yukon	F/F	1	1.45	0:1
Kaspar/Tala	F/M	1	1.45	0:1
Yukon/Tatonga	F/F	2	2.90	1:1
Chitto/Amarok	M/M	2	2.90	1:1
Geronimo/Amarok	M/M	5	7.25	0:5
Amarok/Kay	M/F	1	1.45	0:1
Nanuk/Apache	M/M	2	2.90	2:0
Aragorn/Nanuk	M/M	1	1.45	0:1
Nanuk/Yukon	M/F	2	2.90	2:0
Tala/Una	F/F	2	2.90	1:1
Yukon/Cherokee	F/M	2	2.90	2:0
Geronimo/Apache	M/M	5	7.25	4:1
Apache/Tatonga	M/F	2	2.90	0:2
Tala/Chitto	F/M	3	4.35	1:2
Nanuk/Geronimo	M/M	4	5.80	0:4
Una/Wamblee	F/M	5	7.25	5:0
Yukon/Una	F/F	6	8.70	0:6

*Note*:

^1^—The number of play bows performed by members of the dyad.

^2^—The mean of play bows performed by members of the dyad across all play bows for the sample.

^3^—The relative number of play bows within the dyad by individual. The first value corresponds to the number of play bows performed by the first wolf in the corresponding “dyad” column. The second value corresponds to the number of play bows performed by the second wolf in the corresponding “dyad” column.

### Hypothesis Testing

#### Hypothesis 1: Play bows as visual signals

Hypothesis 1a predicted that play bows would almost always be limited to times when the bower and partner were within one another’s visual field. This prediction was confirmed for dog puppies: for 135 of the 136 play bows the dogs could see one another. In this one exceptional instance, the bower performed an attention-getting behavior by barking as predicted by [11] (Hypothesis 1b).

This prediction was also confirmed for wolf puppies: for all 69 of the play bows the subjects could see one another. Therefore, hypothesis 1b could not be evaluated for the wolves.

#### Hypotheses 2 through 5

**Fig 1:**
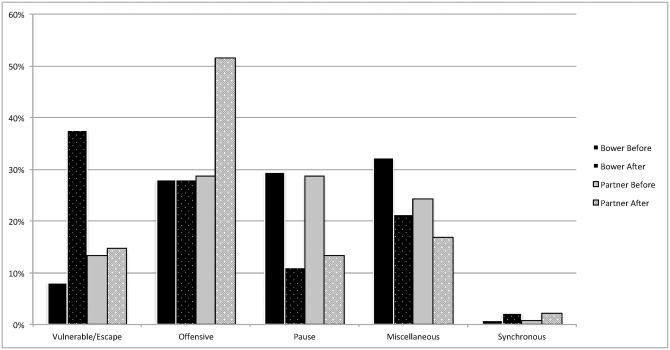
Percentages of Behavior by Timing and Role in Dog Pups. The percentage of behaviors in each behavior categories observed for both the bower and partner before and after the play bow in dog puppies (n = 544).

**Fig 2:**
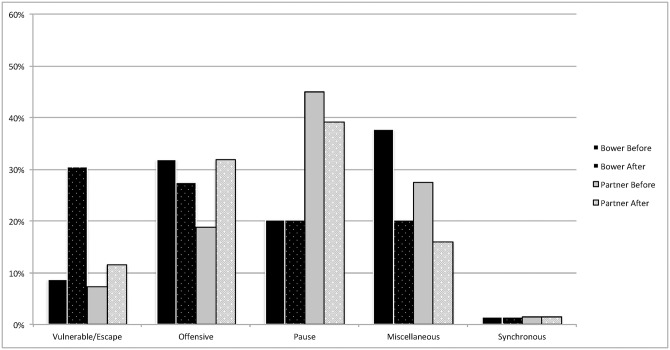
Percentages of Behaviors by Timing and Role in Wolf Pups. The percentage of behaviors in each behavior categories observed for both the bower and partner before and after the play bow in wolf puppies (n = 276).

Percentages for each behavior category were determined before the play bow and after the play bow for both the bower and the partner (see Figs 1 and 2 for dog puppies and wolf puppies respectively). All tests of significance are shown in Table 6 for the dog puppies and Table 7 for the wolf puppies.

**Table 6:** General Linear Mixed Model Results for Dog Puppies.

	Timing		Role	
Behavior Categories	A. Before vs. After for Bower	B. Before vs. After for Partner	C. Bower vs. Partner Before	D. Bower vs. Partner After
1. Pause	**p = .0003**	**p = .0022**	p = 0.8934	p = 0.5774
2. Vulnerable/ Escape	**p < .0001**	p = .7258	p = 0.1723	**p < .0001**
3. Offensive	p = 1.0000	**p = .0002**	p = 0.9592	**p = .0002**
4. Miscellaneous	p = 0.0409	p = 0.1343	p = 0.1542	p = 0.3769
5. Synchronous	p = .3243		p = 1.000	

Note. **“Bold face”**–Significant results

**Table 7:** General Linear Mixed Model Results for Wolf Puppies.

	Timing		Role	
Behavior Categories	A. Before vs. After for Bower	B. Before vs. After for Partner	C. Bower vs. Partner Before	D. Bower vs. Partner After
1. Pause	p = 1.000	p = .4816	**p = 0.0091**	p = 0.0471
2. Vulnerable/Escape	**p = 0.0026**	p = .3869	p = 0.7539	**p = 0.0091**
3. Offensive	p = .5729	p = .0793	p = 0.0793	p = .5729
4. Miscellaneous	p = 0.0265	p = 0.1028	p = 0.3575	p = 0.7042
5. Synchronous	p = 1.000		p = 1.000	

Note. **“Bold face”**–Significant results

#### Hypothesis 2: Clarifying easily misinterpretable behaviors

Hypothesis 2, based on [26], predicted that if play bows function to clarify easily misinterpretable behaviors, bowers should perform more offensive behaviors before and/or after play bows than partners. Results did not confirm this prediction for either species. For both dog and wolf puppies, no difference in offensive behaviors by role was observed before the play bow (Table 6, row 3, column C; Table 7, row 3, column C). Contrary to prediction, after the bow, dog puppy *partners* showed more offensive behaviors than the bower did (Table 6, row 3 column D). No difference in offensive behaviors by timing was observed before the bow for either dog or wolf puppies (Table 6, row 3, column A; Table 7, row 3, column A). However, dog puppy partners showed more offensive behaviors after the bow than before (Table 6, row 3, column B).

Due to evidence suggesting that play bows serve to clarify easily misinterpreted aggressive behaviors by the bower (specifically those associated with bite-shakes; see Bekoff, 1995), the frequencies of bite-like behaviors (nips and bites, hereafter termed bites; see S1 File for definitions) in association with play bows were analyzed. For both dog and wolf puppies, no bite-shakes immediately preceded or followed a play bow by the bower or the partner.

For dog puppies, bite behaviors were performed only 13.6% of the time, occurring only 73 times out of the 544 behaviors for both the bower and the partner before or after the play bow (39 times by the bower, and 34 times for the partner) (*p* = 0.53). However, while bowers were equally likely to perform bite behaviors before (n = 21) and after (n = 18) the bow (*p* = 0.50), partners were more likely to perform bite behaviors after the bow (n = 27) than before (n = 7) (*p* < 0.01). Of the 276 wolf behaviors recorded for both the bower and the partner before and after the bow, only 28 (10.14%) were bites. Bowers (*p* = 0.02) and partners (*p* = 0.04) were more likely to perform bite behaviors before the bow than after (bower before, n = 13; bower after, n = 7; partner before, n = 6; partner after, n = 2). Bowers (n = 20) were more likely to perform bite behaviors, in general, than partners (n = 8) (*p* < 0.01).

#### Hypothesis 3: Attacking & escaping the play partner

Hypothesis 3a predicted that play bows might function to better position the bower to ‘attack’ the play partner. If so, bowers should perform more offensive behaviors after the play bow than before. As noted above (Hypothesis 2), bowers, in both dog and wolf puppy samples were not more likely to perform offensive behaviors after the bow than before (Table 6, row 3 column A; Table 7, row 3 column A). In fact, after the play bow, dog puppy partners rather than bowers were more likely to perform offensive behaviors (Table 6, row 3 column D). For wolf puppies, this difference was not observed, as no differences were observed in offensive behaviors between wolf puppy play bowers and partners (Table 7, row 3 columns D).

Hypothesis 3b alternatively suggested that play bows might function to better position the bower to escape from the play partner. If so, bowers would be expected to perform more vulnerable/escape behaviors after the play bow than before. The prediction for hypothesis 3b was confirmed for both dog and wolf puppies: bowers performed more vulnerable/escape behaviors after the play bow than before (Table 6, row 2 column A; Table 7, row 2 column A). Additionally, both dog and wolf puppy play bowers were also more likely to perform vulnerable/escape behaviors after the play bow than were their play partners (Table 6, row 2 column D; Table 7, row 2 column D), an effect of role consistent with hypothesis 3 but not predicted by it.

#### Hypothesis 4: Re-initiation of play

Hypothesis 4 predicted that both the bower and the partner would show more pause behaviors before the play bow than after. To test this hypothesis, the effects of timing (before *versus* after the play bow) were compared for proportions of pause behavior by both the bower and the partner. Results confirmed the predicted effect for dog puppies (Table 6, row 1, columns A and B). Both the bower and the partner displayed more pause behaviors before the play bow than after. Results did not confirm the prediction for wolf puppies (Table 7, row 1, columns A and B). However, wolf partners were significantly more likely to show a pause behavior before the play bow than was the bower (Table 7, row 1, column C); a difference that was not significant for the dog puppies.

#### Hypothesis 5: Play bow synchronization

Hypothesis 5 predicted that if play bows help a pair to synchronize behaviors, then synchronous behaviors should be more common after a play bow than before. Since, by definition, the proportion of synchronous behaviors must always be the same for the bower and the partner, one statistical test applies to both roles. The prediction was not confirmed for either dog or wolf puppies: bowers and partners were no more likely to perform synchronous behaviors after the play bow than before (Table 6, row 5, column A; Table 7, row 5, column A).

## Discussion

In order to address the function of the play bow within wolf and dog puppy dyadic play we analyzed the behaviors occurring immediately before and after a bow for both the play bower and play partner. In particular, our aim was to evaluate a variety of proposed hypotheses derived from the literature.

Our results suggest that dog and wolf puppy play bowers, like adult dogs, use play bows as a visual signals [11,24]. Almost all bows were performed when the dyad was visually attentive to one another, suggesting that dog and wolf puppy play bowers, like adult dogs, may understand the context in which it is appropriate to use the visual signal. Moreover, the single play bow in dog puppies that occurred without visual contact also included an attention-getting behavior in the form of a bark, providing proof of concept that dog puppies may understand when to use attention-getting behaviors in association with a bow. Future research should be conducted to determine the age at which awareness of the partner’s attentional state occurs and how it develops over time.

No evidence was found in the GLMM to suggest that play bows in dog and wolf puppies function as a means to clarify easily misinterpretable behaviors. Play bows did not emphasize playful intent during instances in which the bower’s behaviors were most susceptible to misinterpretation (Table 8). When behavior categories were analyzed for dog puppies, behaviors shown after play bows differed by role but in the opposite direction to that expected by Bekoff [26] results: *partners* showed offensive behaviors more often after the bow than bowers did, whereas neither bowers nor partners showed any difference in the proportions of this behavior category before the bow. For the wolf puppies, when behavior categories were analyzed, no difference was found as a function of either role or timing. Furthermore, no bite-shakes were ever observed in either species, and bite behaviors, such as nips and bites, occurred at low rates. For dog puppies, bowers and partner were equally likely to perform bite behaviors, and partners rather than bowers were more likely to perform bite behaviors after the bow than before. However, wolf puppy play bowers performed more bite behaviors than their partners. Furthermore, bowers and partners were more likely to perform bite behaviors before the bow than after. Therefore, in a handful of instances, wolf puppy bowers may perform play bows after bite behaviors, potentially to clarify the misinterpretability of the signal. However, these results may simply be an effect of the tendency for the wolf puppy play bowers to show more play activity than their partners did.

**Table 8:** Summary of evidence for hypotheses 1–5 in wolf puppies, dog puppies, and adult dogs.

Hypothesis	Wolf Puppies	Dog Puppies		Adult Dogs
1: Play bows as visual signals	**Yes**	Yes*	Yes*	
2: Clarification of easily misinterpretable behaviors	Maybe	No*	No*	
3a: Attacking the play partner	No	No*	No*	
3b Escaping from the play partner	**Maybe**	Maybe*	Maybe*	
4: Re-initiation of play	No	Yes*	Yes*	
5: Play bow synchronization	No	No	Yes	

Note.–Adult dog results are from Byosiere et al. [24]

**“Bold face”**–Results consistent across samples

*—Results consistent across young and adult dogs

These findings contrast with those of Bekoff [26], but it is worth emphasizing that the age of the subjects observed may be one reason for the differences. The dog and wolf puppies in Bekoff’s study were all younger than any of those observed in this study (3–7 weeks old compared to approximately 2–8 months). Bekoff’s dog sample intermixed 3–7 week old puppies with adult dogs without information on how many observations each accounted for. Without this information we cannot say how likely it is that differences between Bekoff’s results and ours are due to differences in the ages of the wolf and dog puppies in each study, so we simply note it as a possibility. However, the results reported here are consistent with Byosiere et al. [24], who found that play bows did not function to clarify easily misinterpretable behaviors in adult dogs (Table 8).

No support was found to indicate that play bows function strategically to better position the bower to attack the play partner; however mixed results were observed in regard to escaping the play partner (Table 8). In the dog puppies, play *partners* rather than bowers, showed significantly higher proportions of *offensive behaviors* after a play bow than before. In the wolf puppies, role (bower or partner) did not affect proportions of offensive behaviors shown. While bowers were more likely to perform vulnerable/escape behaviors after the play bow than before, our results indicate there was not a higher proportion of offensive behaviors to escape from. Although the latter remains a possibility for a small number of interactions, a more parsimonious explanation is that play bows function to reinitiate play rather than serving as a maneuver to escape the play partner. These findings in both dog and wolf puppies are consistent with those observed in adult dogs [24].

Play bows in dog puppies most often functioned to stimulate action after a pause in play. Specifically, bowers and partners showed proportionately more active behaviors after the play bow than before. These results replicate Byosiere et al. [24] findings for adult dog play, and are in line with Horowitz [11] who observed that play signals occurred more frequently after pauses (Table 8). In wolf puppy play, bowers and partners were no more likely to be in a stationary position before the play bow than after (Table 8). However, wolf puppy play bowers were more likely to perform vulnerable/escape behaviors after the bow than before and compared to the partner. These results suggest that wolf puppy play bowers may be performing bows with intent similar to that observed in dogs. However, while wolf partners were more likely to perform pause behaviors before the bow than bowers, they were no more likely to adopt active roles after the bow than before. Therefore it appears that the play signal, while performed similarly across canids, failed to entice wolf play partners into engaging in play.

While these results indicate that play bows may function differently in wolf and dog puppies with regard to re-initiating play, further study is needed to determine if this finding represents a genuine species difference or is explicable by factors not accounted for in this study. These differences could be attributed to several alternative factors based on when the videos were recorded. For example, the time spent playing, outside temperature, time of day, or whether the individuals had just eaten could also account for the differences observed between dog and wolf puppies. Additionally, demographic differences in the composition of the puppy groups, such as in total size, the number of dyads, the number of littermates, and the ratio of males to females may also have contributed to the difference.

Finally, in both dog and wolf puppy samples play bowers and partners were observed performing very few synchronous behaviors (Figs 1 and 2). These results are contrary to those found in adult dog play interactions [24]. In such dogs, play bows were often followed by synchronous actions. It seems likely that age effects on play style explain this difference. Mutual rear-ups occurred only once (once for the bower and partner) in dog puppies, and never in wolf puppies. Therefore, it is possible that the puppies were limited by their lack of bodily control or social experience to rear-up or align their behaviors with a partner, and that this behavior increases in frequency with age, or potentially as a function of a preferred play partner rather than a littermate [27,30,33].

Taken together, findings from this study and the previous study on adult dogs [24] suggest that play bows do not occur at random and do not, therefore, simply enhance the play atmosphere in a general way. Instead, their association with particular behaviors before and after the play bow suggests strategic use of this play signal to accomplish immediate goals [9,27], including continuation of play by enticing the partner into a runaway/chase interaction. However, while many of our findings are similar across dog and wolf puppies, and consistent with those observed in adult dogs, species differences may exist. Whether these reflect confounds, behavioral patterns specific to our sample, or species differences due to domestication requires further investigation.

## Supporting Information

S1 File Ethogram, behavioral codes, and definitions. (PDF)

S2 File GLMM analysis output for both dog and wolf puppies. (PDF)

S1 Table Determinants of Visual Signals. The bower and the partner were considered to be within each other’s field when the majority of the front torso was facing towards the play partner. Players were considered to be not facing one another when more than half of each individual’s front torso was facing away from the other.
(PDF)
