# Supplementary material for: Investigating the Function of Play Bows in Dog and Wolf Puppies (Canis lupus familiaris, Canis lupus occidentalis)
Source: PLoS One. 2016 Dec 29;11(12):e0168570. doi: 10.1371/journal.pone.0168570 (PMC5199004; doi:10.1371/journal.pone.0168570)
Supplement: S1 File — (PDF) [file pone.0168570.s001.pdf]

S1.1. Steps versus strides

Within walking movements, individuals can run in strides or walk in steps. However, definitions between strides and steps may not be mutually exclusive. In order to counteract this problem we incorporated a definitional difference between steps and strides. Steps are considered to be a one-paw movement, where an individual lifts one leg and then places it down, not in tandem with lifting the opposite leg. These steps are most commonly seen when the individual is walking. Strides, however are involved in running movements or when galloping occurs. A stride is considered to occur when both the individual's paws lift from the ground in tandem while in motion. The paws do not need to move simultaneously, however, when one paw is off the ground, the other should be as well.

S1.2. Ethogram

Table 1

Ethogram of behavior codes organized by behavior categories

Vulnerable/ Escape	Offensive	Synchronous	Miscellaneous	Pause
Receives bite	Bites move	Mutual rear up	Close approach	Relatively stationary
	Bites still	Move together	Chin-over move	Sitting
Receives muzzle bite	Bites muzzle		Chin-over stationary	Lying down
Runaway	Charge		Close non directional movement	
	Chase		Far non directional movement	
Being forced down	Force down		Move away	
Receives genital sniff	Gives genital sniff		Partial approach	

Receives mount	Mount	Play bow
Receives nip	Nip move	Play face
	Nip still	Out of view
Receives push/tackle	Push/tackle	
	Failed tackle	
Voluntary down		
Gives muzzle lick	Receives muzzle Lick	
Receives overs during downs	Overs during downs	

S1.2.1. Vulnerable/escape behaviors

- Receive bite (RB): Y is bitten by X, with their jaws maintaining contact with Y's fur or skin for at least 1/2 second. Code X as either stationary or in motion, however, it is not required for Y. Enter in the "comments" section whether they bite on the muzzle ("muzzle") or other part of the body ("body").
- Receive nip (RN): Y is nipped by X, with their jaws making two or more distinct bite nibbles on Y's fur or skin. Code X as either stationary or in motion, however, it is not required for Y. Enter in the "comments" section whether they bite on the muzzle ("muzzle") or other part of the body ("body").
- Receive overs during downs (RODD): Y receives X standing over or lying on Y with at least 50% of X's torso over Y's torso (or vice versa: 50% of L's torso is under X's torso), or X sits and exerts weight directly on Y's head or torso with a distinct pause in the sitting position
- Runaway (RA): Y moves at least five running strides away from X. "Running strides" means that front legs are moving in unison (i.e. "galloping") at a pace of at least two strides (each time front paws hit the ground) per second. They must

be within three meters of each other at the start of the behavior. Simultaneously, X may remain “relatively stationary”, approach, or move away from Y.

- Being forced down (BFD): Y falls to the ground, seemingly caused by a forceful move by X (see “forced down”) on the part of X (with any part of their body). If X is coded as forced down, and Y follows the behavioral trajectory of being forced down, code Y as BFD.
- Receive genital sniff (RGS): Y holds hind legs apart while in belly-up position on the ground to allow X to put snout on or near Y's genitals for an investigatory sniff.
- Receive muzzle bite (RMB): by not pulling away or engaging in mutual muzzle biting, Y allows X to maintain a position in which X's open mouth is around Y's closed muzzle; X's bite is clearly inhibited and the position is not maintained owing to the force of the bite. Both individuals are in a relatively stationary position (standing, sitting, or lying down), and must maintain the bite for at least one second.
- Receive muzzle lick (RML): Y licks X on or around X’s mouth and/or muzzle, Y’s tongue must make contact with X’s mouth and/or muzzle.
- Receives push/tackle (RPT*)*: Y is touched with forceful contact by X due to rearing up and placing one or both front paws on Y; forelimbs may or may not be wrapped around Y's body in a tackle grasp (if it results in a down, code as forced down instead of receives push/tackle).

- Give muzzle lick in movement (LM): Y licks on or around X's mouth and/or muzzle, Y's tongue must make contact with X's mouth and/or muzzle. Y should be walking/running simultaneously for at least two steps.
- Give muzzle lick while stationary (LS): Y licks on or around X’s mouth and/or muzzle, Y’s tongue must make contact with X’s mouth and/or muzzle. Y should be stationary for at least two seconds.
- Voluntary down (VD): Y drops partially or completely to the ground without X's enforcement; Y must be actively interacting with X at the moment the down is recorded. If Y occupies a lying down position where all paws make contact with the ground then code as lying down. However, if a lying down position is followed by Y’s paws being in the air within ½ a second after the play bow, code as a voluntary down.

S1.2.2. Offensive behaviors

- Bites movement (BM): X bites Y, with their jaws maintaining contact with Y’s fur or skin for at least 1/2 second. X must be moving simultaneously, for at least two steps (defined as movement of one front paw). Enter in the “comments” section whether they bite on the muzzle (“muzzle”) or other part of the body (“body”).
- Bites still (BS): X bites Y, with their jaws maintaining contact with Y’s fur or skin for at least 1/2 second. Both individuals must be standing, laying down, or sitting position while fulfilling the criteria for relatively stationary: Individual is standing still for at least 1.5 seconds before or after the PB. Individual may move

somewhat, but not for more than two strides. Enter in the “comments” section whether they bite on the muzzle (“muzzle”) or other part of the body (“body”).

- Muzzle Bite (MB): X bites Y in a stationary position (standing, sitting, or lying down), and must maintain the bite for at least one second. Y allows X to maintain a position in which X's open mouth is around Y's closed muzzle; X's bite is clearly inhibited and the position is not maintained owing to the force of the bite.
- Chase (CH): X moves at least five running strides toward Y. “Running strides” means that front legs are moving in unison (i.e. “galloping”) at a pace of at least two strides per second. Simultaneously, Y may approach, or move away from X but if Y remains relatively stationary then code as “charge.” Individuals must be within three meters of each other at the conclusion of X’s action. There may be contact, but if contact fulfills the requirements for tackle, failed tackle, or chin over, code that instead.
- Charge (CG): X moves at least five running strides toward Y. “Running strides” means that front legs are moving in unison (i.e. “galloping”) at a pace of at least two strides per second. Simultaneously, Y remains “relatively stationary.” Individuals must be within three meters of each other at the conclusion of X’s action. There may be contact, but if contact fulfills the requirements for tackle, failed tackle, or chin over, code that instead.
- Forced down (FD): X uses sufficient physical force to cause Y to drop partially or completely to the ground; force may be applied with a bite, push/tackle, body or some other forceful movement

- Gives genital sniff (GS): X is allowed to put snout on or near Y's genitals for an investigatory sniff. Y holds hind legs apart while in belly-up position on the ground.
- Nip Movement (NM): X bites Y, with their jaws making two or more distinct bite nibbles on Y’s fur or skin. X must be moving toward Y simultaneously, for at least two steps (defined as movement of one front paw). Enter in the “comments” section whether they bite on the muzzle (“muzzle”) or other part of the body (“body”).
- Nip Still (NS): X bites Y, with their jaws making two or more distinct bite nibbles on Y’s fur or skin. Both individuals must be standing, laying down, or sitting position while fulfilling the criteria for relatively stationary: Individual is standing still for at least 1.5 seconds before or after the play bow. Individual may move somewhat, but not for more than two strides. Enter in the “comments” section whether they bite on the muzzle (“muzzle”) or other part of the body (“body”).
- Mount (MO): X rears up to place forelegs on Y's back in a front, lateral or rear mount position, differentiated from push/tackles by X's rounded spine position during the mount and a distinct pause in the stable mount position, which may or may not be accompanied by thrusting
- Overs during downs (ODD): X stands over or lies on Y with at least 50% of X's torso over Y's torso (or vice versa: 50% of Y's torso is under X's torso), or X sits and exerts weight directly on L's head or torso with a distinct pause in the sitting position

- Push/tackle (PT*)*: X rears up and places one or both front paws on Y with forceful contact; forelimbs may or may not be wrapped around Y's body in a tackle grasp (if it results in a down, code as forced down instead of independent push/tackle).
- Failed tackle (FT): X rears up as if to do a push/tackle, but is unable to make contact with Y with either paw, because Y quickly moves out of the way, or is already moving.

S1.2.3. Synchronous behaviors

Synchronous behaviors include two pre-defined behavioral states, mutual rear-ups and move together, that both players displayed one of these two behaviors in unison. Note that synchronous behaviors must be equal, as both individuals need to be performing them.

- Mutual Rear Up (MRU): X and Y rear up simultaneously and X or Y or X and Y place their paws on the other (note: X may be somewhat forced to keep their paws below the Y, therefore not actually touching Y. However, as long as both front paws from both Y and X leave the ground, code both X and Y as a mutual rear up). If only one individual rears up code as Push/Tackle.
- Movement together (MT): Movement by X that is not really directed toward or away from the Y (e.g. both running or walking side-by-side). X and Y must move at least two steps, and more or less maintain distance between them that is seen during the play bow before or after, staying within three meters of each other.

S1.2.4. Miscellaneous behaviors

- Movement away (MA): Y moves at least two steps away from X. They must be within three meters of each other at the start of the behavior. Simultaneously, X may remain “relatively stationary”, approach, or move away from Y. NOTE: BEHAVIOR SHOULD NOT FIT CRITERIA FOR “RUN AWAY”.
- Partial approach (PA): X moves at least two steps toward Y. Simultaneously, Y may stay “relatively stationary”, approach, or move away from X. Individuals must be between one and three meters of each other at the conclusion of X’s action for it to be considered a partial approach. There may be contact, but if contact fulfills the requirements for tackle, failed tackle, or chin over, code that instead. NOTE: BEHAVIOR SHOULD NOT FIT CRITERIA FOR “CHARGE/CHASE”.
- Close approach (CA): X moves at least two steps (defined as movement of one front paw) toward Y. Simultaneously, Y may stay “relatively stationary”, approach, or move away from X. Individuals must be within one meter of each other at the conclusion of X’s action for it to be considered a close approach. There may be contact, but if contact fulfills the requirements for tackle, failed tackle, or chin over, code that instead. NOTE: BEHAVIOR SHOULD NOT FIT CRITERIA FOR “CHARGE/CHASE”.
- Chin-over movement (CM): X places chin over Y’s back, usually right behind the neck or near Y’s shoulders; X’s chin may or may not touch Y but must be at or near a 90-degree angle in relation to the plane of Y’s spine. In chin-over movement both individuals must be in movement for at least two steps. Chin-over must last for at least one second.

- Chin-over stationary (CS): X places chin over Y's back, usually right behind the neck or near Y's shoulders; X's chin may or may not touch Y but must be at or near a 90-degree angle in relation to the plane of Y's spine. In chin over stationary both individuals must be fulfill criteria for relatively stationary: Individual is standing still for at least 1.5 seconds before or after the play bow. Individual may move somewhat, but not for more than two strides. X must engage in the behavior for at least ½ of a second.
- Close Non-Directional Movement (CNM): 'Bouncy' or 'spastic' movement, in which X moves at least two steps, but not in any particular direction. For example, X may take one step toward Y, and one step away, or vice versa. X may also move laterally, with some characteristics of a play bow, but without completely fulfilling the play bow definition (e.g. rear not raised above front, or lasting less than a third of a second). Close non-directional movement occurs when X and Y are closer than three meters apart for the entirety of the action.
- Far Non-Directional Movement (FNM): 'Bouncy' or 'spastic' movement, in which X moves at least two steps, but not in any particular direction. For example, X may take one step toward Y, and one step away, or vice versa. X may also move laterally, with some characteristics of a play bow, but without completely fulfilling the play bow definition (e.g. rear not raised above front, or lasting less than a third of a second). Any movement of two steps or more that occurs when X is more than three meters apart for the entirety of the action.

- Play bow (PB): Can be coded as behaviors before/after for either X or Y. Same criteria apply as described in the play bow definition, but length of play bow and secondary behaviors do not need to be coded.
- Play face (PF) – Y engages X by using an open mouth in tandem with a back and forth, or side to side motion around X. Can be accompanied with any other action such as relatively stationary, close approach, close-non directional movement, etc. Teeth may be showing. No amount of steps is required for play face to occur as long as the play face lasts ½ a second by both L and W, or one of the two.

S1.2.5. Pause behaviors

- Relatively Stationary (RS): X is standing still for at least 1.5 seconds before or after the play bow. X may move somewhat, but not for more than two strides.
- Sitting (ST): X is sitting down for at least 1.5 seconds before or after the play bow.
- Lying down (LD): X is lying down for at least 1.5 seconds before or after the play bow. If X's legs are in the air within ½ a second after the play bow code as a voluntary down.
