# Supplementary material for: Investigating the Function of Play Bows in Dog and Wolf Puppies (Canis lupus familiaris, Canis lupus occidentalis)
Source: PLoS One. 2016 Dec 29;11(12):e0168570. doi: 10.1371/journal.pone.0168570 (PMC5199004; doi:10.1371/journal.pone.0168570)
Supplement: S2 File — (PDF) [file pone.0168570.s002.pdf]

Table S2File 1

Tests of Fixed Effects in Dog Puppies

Pause	Effect	Numerator *df*	Denominator *df*	*F* Value	Pr > *F*
	Role	1	496	0.14	0.7080
	Timing	1	496	22.76	<.0001
	Timing*Role	1	496	0.28	0.5956
Vulnerable/Escape	Role	1	496	1.97	0.1613
	Timing	1	496	16.67	<.0001
	Timing*Role	1	496	12.92	0.0004
Offensive	Role	1	496	7.03	0.0083
	Timing	1	496	6.89	0.0090
	Timing*Role	1	496	6.89	0.0090
Miscellaneous	Role	1	496	2.55	0.1112
	Timing	1	496	6.20	0.0131

	Timing*Role	1	496	0.08	0.7810
Synchronous	Role	1	496	0.00	1.0000
	Timing	1	496	1.95	0.1637
	Timing*Role	1	496	0.00	1.0000

Table S2File 2

*Timing*Role Least Squares Means in Dog Puppies*

	Timing	Role	Estimate	Standard Error	*df*	*t* Value	Pr > |*t*|
Pause	Before	Bower	-0.8946	0.2026	496	-4.41	<.0001
	Before	Partner	-0.9305	0.2040	496	-4.56	<.0001
	After	Bower	-2.1138	0.2842	496	-7.44	<.0001
	After	Partner	-1.9055	0.2642	496	-7.21	<.0001
Vulnerable/Escape	Before	Bower	-2.4286	0.3261	496	-7.45	<.0001
	Before	Partner	-1.8754	0.2673	496	-7.02	<.0001

	After	Bower	-0.4908	0.1969	496	-2.49	0.0130
	After	Partner	-1.7521	0.2569	496	-6.82	<.0001
Offensive	Before	-0.8798	0.2167	496	-4.06	<.0001	-0.8798
	Before	-0.8659	0.2155	496	-4.02	<.0001	-0.8659
	After	Bower	-0.8798	0.2167	496	-4.06	<.0001
	After	Partner	0.1143	0.2005	496	0.57	0.5687
Miscellaneous	Before	Bower	-0.7904	0.2100	496	-3.76	0.0002
	Before	Partner	-1.1824	0.2255	496	-5.24	<.0001
	After	Bower	-1.3633	0.2334	496	-5.84	<.0001
	After	Partner	-1.6402	0.2516	496	-6.52	<.0001
Synchronous	Before	Bower	-5.9938	1.1691	496	-5.13	<.0001
	Before	Partner	-5.9938	1.1691	496	-5.13	<.0001
	After	Bower	-4.8242	0.8226	496	-5.86	<.0001
	After	Partner	-4.8242	0.8226	496	-5.86	<.0001

Table S2File 3

*Tests of Effect Slices for Timing*Role Sliced By Role in Dog Puppies*

Pause	Role	Numerator *df*	Denominator *df*	*F* Value	Pr > *F*
	Bower	1	496	13.40	0.0003
	Partner	1	496	9.45	0.0022
Vulnerable/Escape	Bower	1	496	28.60	<.0001
	Partner	1	496	0.12	0.7258
Offensive	Bower	1	496	0.00	1.0000
	Partner	1	496	14.54	0.0002
Miscellaneous	Bower	1	496	4.20	0.0409
	Partner	1	496	2.25	0.1343
Synchronous	Bower	1	496	0.97	0.3243
	Partner	1	496	0.97	0.3243

Table S2File 4

*Tests of Effect Slices for Timing*Role Sliced By Timing in Dog Puppies*

	Timing	Numerator *df*	Denominator *df*	*F* Value	Pr > *F*
Pause	Before	1	496	0.02	0.8934
	After	1	496	0.31	0.5774
Vulnerable/Escape	Before	1	496	1.87	0.1723
	After	1	496	17.48	<.0001
Offensive	Before	1	496	0.00	0.9592
	After	1	496	14.59	0.0002
Miscellaneous	Before	1	496	2.04	0.1542
	After	1	496	0.78	0.3769
Synchronous	Before	1	496	0.00	1.0000
	After	1	496	0.00	1.0000

Table S2File 5

Tests of Fixed Effects in Wolf Puppies

Pause	Effect	Numerator *df*	Denominator *df*	*F* Value	Pr > *F*
	Role	1	232	10.18	0.0016
	Timing	1	232	0.20	0.6539
	Timing*Role	1	232	0.20	0.6539
Vulnerable/Escape	Role	1	232	3.24	0.0733
	Timing	1	232	6.86	0.0094
	Timing*Role	1	232	1.67	0.1980
Offensive	Role	1	232	0.82	0.3658
	Timing	1	232	0.82	0.3658
	Timing*Role	1	232	2.81	0.0953
Miscellaneous	Role	1	232	0.75	0.3871
	Timing	1	232	7.38	0.0071
	Timing*Role	1	232	0.09	0.7634
Synchronous	Role	1	232	0.00	1.0000
	Timing	1	232	0.00	1.0000
	Timing*Role	1	232	0.00	1.0000

Table S2File 6

*Timing*Role Least Squares Means in Wolf Puppies*

	Timing	Role	Estimate	Standard Error	*df*	*t* Value	Pr > |*t*|
Pause	Before	Bower	-1.3194	0.3452	232	-3.82	0.0002
	Before	Partner	-0.2635	0.2987	232	-0.88	0.3786
	After	Bower	-1.3194	0.3452	232	-3.82	0.0002
	After	Partner	-0.5126	0.3026	232	-1.69	0.0916
Vulnerable/Escape	Before	Bower	-2.3514	0.4272	232	-5.50	<.0001
	Before	Partner	-2.5494	0.4644	232	-5.49	<.0001
	After	Bower	-0.8267	0.2616	232	-3.16	0.0018
	After	Partner	-2.0314	0.3760	232	-5.40	<.0001
Offensive	Before	Bower	-0.7973	0.2839	232	-2.81	0.0054
	Before	Partner	-1.5126	0.3307	232	-4.57	<.0001

	After	Bower	-1.0104	0.2945	232	-3.43	0.0007
	After	Partner	-0.7973	0.2839	232	-2.81	0.0054
Miscellaneous	Before	Bower	-0.5504	0.2762	232	-1.99	0.0475
	Before	Partner	-0.8966	0.2945	232	-3.04	0.0026
	After	Bower	-1.4223	0.3234	232	-4.40	<.0001
	After	Partner	-1.5944	0.3493	232	-4.56	<.0001
Synchronous	Before	Bower	-4.6394	1.1572	232	-4.01	<.0001
	Before	Partner	-4.6394	1.1572	232	-4.01	<.0001
	After	Bower	-4.6394	1.1572	232	-4.01	<.0001
	After	Partner	-4.6394	1.1572	232	-4.01	<.0001

Table S2File 7

*Tests of Effect Slices for Timing*Role Sliced By Role in Wolf Puppies*

Pause	Role	Numerator *df*	Denominator *df*	*F* Value	Pr > *F*
	Bower	1	232	0.00	1.0000

	Partner	1	232	0.50	0.4816
Vulnerable/Escape	Bower	1	232	9.26	0.0026
	Partner	1	232	0.75	0.3869
Offensive	Bower	1	232	0.32	0.5729
	Partner	1	232	3.11	0.0793
Miscellaneous	Bower	1	232	4.99	0.0265
	Partner	1	232	2.68	0.1028
Synchronous	Bower	1	232	0.00	1.0000
	Partner	1	232	0.00	1.0000

Table S2File 8

*Tests of Effect Slices for Timing*Role Sliced By Timing in Wolf Puppies*

	Timing	Numerator *df*	Denominator *df*	*F* Value	Pr > *F*
Pause	Before	1	232	6.92	0.0091
	After	1	232	3.98	0.0471

Vulnerable/Escape	Before	1	232	0.10	0.7539
	After	1	232	6.92	0.0091
Offensive	Before	1	232	3.11	0.0793
	After	1	232	0.32	0.5729
Miscellaneous	Before	1	232	0.85	0.3575
	After	1	232	0.14	0.7042
Synchronous	Before	1	232	0.00	1.0000
	After	1	232	0.00	1.0000
