# Supplementary material for: Investigating the Function of Play Bows in Dog and Wolf Puppies (Canis lupus familiaris, Canis lupus occidentalis)
Source: PLoS One. 2016 Dec 29;11(12):e0168570. doi: 10.1371/journal.pone.0168570 (PMC5199004; doi:10.1371/journal.pone.0168570)
Supplement: S1 Table — The bower and the partner were considered to be within each other’s field when the majority of the front torso was facing towards the play partner. Players were considered to be not facing one another when more than half of each individual’s front torso was facing away from the other. (PDF) [file pone.0168570.s003.pdf]

Stance Occupied	Facing Each Other?
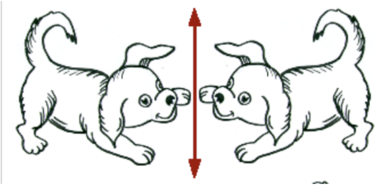	Yes
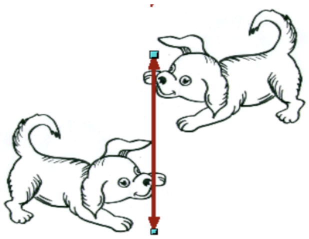	Yes
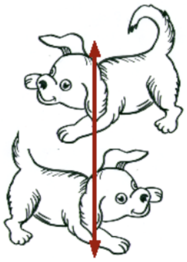	No

Note: Drawing from http://erwinnavyanto.in/cartoon-dog-drawings-in-pencil/
